# A fecal sequel: Testing the limits of a genetic assay for bat species identification

**DOI:** 10.1371/journal.pone.0224969

**Published:** 2019-11-14

**Authors:** Faith M. Walker, Abby Tobin, Nancy B. Simmons, Colin J. Sobek, Daniel E. Sanchez, Carol L. Chambers, Viacheslav Y. Fofanov

**Affiliations:** 1 Bat Ecology & Genetics Lab, School of Forestry, Northern Arizona University, Flagstaff, Arizona, United States of America; 2 Pathogen and Microbiome Institute, Northern Arizona University, Flagstaff, Arizona, United States of America; 3 Department of Mammalogy, Division of Vertebrate Zoology, American Museum of Natural History, New York, New York, United States of America; 4 School of Informatics, Computing, and Cyber Systems, Northern Arizona University, Flagstaff, Arizona, United States of America; University of Guelph, CANADA

## Abstract

DNA metabarcoding assays are powerful tools for delving into the DNA in wildlife feces, giving unprecedented ability to detect species, understand natural history, and identify pathogens for a range of applications in management, conservation, and research. Next-generation sequencing technology is developing rapidly, which makes it especially important that predictability and reproducibility of DNA metabarcoding assays are explored together with the post-depositional ecology of the target taxon’s fecal DNA. Here, we defined the constraints of an assay called ‘Species from Feces’ used by government agencies, research groups, and non-governmental organizations to identify bat species from guano. We tested assay sensitivity by examining how time and humidity affect the ability to recover and successfully sequence DNA in guano, assessing whether a fecal pellet from a rare bat species could be detected in a background of feces from other bat species, and evaluating the efficacy of Species from Feces as a survey tool for bat roosts in temperate and tropical areas. We found that the assay performs well with feces over two years old in dry, cool environments, and fails by 12 months at 100% relative humidity. We also found that it reliably identifies rare DNA, has great utility for surveying roosts in temperate and tropical regions, and detects more bat species than do visual surveys. We attribute the success of Species from Feces to characteristics of the assay paired with application in taxa that are particularly well-suited for fecal DNA survival. In a time of rapid evolution of DNA metabarcoding approaches and their use with feces, this study illustrates the strengths and limitations of applied assays.

## Introduction

Fecal pellets can be considered the new “gold nuggets” of wildlife biology because of the insight they provide into biodiversity, diet, and disease without having to trap, handle, or even observe the animals that produce them [1–3]. Despite the universality and availability of feces and the relative ease of collecting this type of sample, only recently has it been possible to fully exploit fecal DNA. Previous challenges of working with characteristically degraded fecal DNA [4] have been overcome with the maturation and cost-effectiveness of second-generation sequencing and analysis [5–8]. DNA metabarcoding via next-generation amplicon sequencing allows simultaneous identification of taxa through parallel sequencing of thousands of DNA molecules present in a sample, taking advantage of short DNA barcodes that are targeted using universal primers [7]. The sensitivity of this approach means that sample types can be used that were formerly problematic due to low DNA quantity. Wildlife geneticists are taking advantage of feces in a next-generation sequencing context, as evidenced by a doubling of the number of papers published in 2018 compared to the previous year (17 and 8, respectively; Web of Science, basic search on topic using two search fields: “feces” and “DNA metabarcoding”, and “faeces” and “DNA metabarcoding”).

However, there are a number of considerations when collecting feces for genetic analysis. After deposition, wildlife feces are exposed to environmental conditions over a period of time prior to collection, which can accelerate degradation of DNA that is already compromised. Successful sequencing has been found to be higher for fresher feces [9, 10], and for samples collected in conditions such as cold temperatures [11, 12], dry climates with protection from precipitation [13], and low UV exposure [14]. Further, DNA response to these variables has been found to be taxon-specific (e.g., accurate genotyping after 2 months in feral horses [15], errors after 7 days in coyotes [16]) and target-specific (from bat guano: change in bacterial community composition after 1 hour [17], identification of bat species at 3 months [3]). DNA concentration can also vary by collection/preservation method [15, 18] and by the order the feces traveled through the intestines [19]. Hence, the pairing of rapidly evolving genetic sequencing methods with fecal-derived DNA means it is important that the constraints inherent to particular fecal types and their associated assays are explored and delineated to allow for predictability and reproducibility [20, 21].

We developed a DNA mini-barcode assay called ‘Species from Feces’ that uses next-generation amplicon sequencing to identify the bat species that contributed to a pooled guano sample consisting of up to 200 fecal pellets collected from a roost [3]. The assay is effective for identification of bat species worldwide (92% to species level and 8% to genus level for taxa with publically available COI barcodes; searchable database [22]). Since its publication in 2016, at least 80 research and/or management groups on four continents (North America, Africa, Europe, and Asia) have employed the assay to identify bat species that used roosts such as caves, mines, buildings, bridges, trees, crevices, and bat boxes. Users have included U.S. state and federal agencies (e.g., the National Park Service, Forest Service, Bureau of Land Management, state wildlife management departments, Departments of Transportation, Department of Agriculture, Department of Defense), non-governmental organizations, environmental consulting firms, and universities [23–25]. This assay, in addition to identifying bat species that use particular roosts [25], has also been employed to confirm visual identifications of captured bats [23], identify bat carcasses at windfarms, screen fertilizer to determine the species that contributed guano, detect nectar-feeding bats from saliva on agave flowers, and evaluate the effects of gates on bat use of mines [24].

As applications for the assay become more varied, questions have arisen regarding laboratory and field limitations of the assay. Such questions include: Can the Species from Feces assay detect a bat species that contributes only a single guano pellet to a pooled guano sample of other bat species?; and, Can guano stored over long periods (“old”) or guano at room temperature yield sufficient DNA for identification? To assist the conservation and resource management community with predictability of the assay and the research community with reproducibility, we sought a better understanding of the post-depositional viability of bat fecal DNA, the sensitivity and specificity of the assay, and the utility of genetic surveys for bats in temperate and tropical regions.

To this end, we performed a series of lab and field tests to address four questions: 1) How do time and humidity impact the ability to successfully Sanger sequence DNA from bat feces? To address this question, we tested DNA from guano of known age in a variety of locations (caves, office) with differing relative humidity and temperature, over 2.5 years; 2) How rare can feces be in a sample for DNA to still be detected? Our goal was to determine whether the assay is capable of accurately detecting a rare bat species in the DNA background of multiple other bat species. To assess the sensitivity (ability to detect rare DNA) and specificity (presence of false positives or negatives) of the Species from Feces DNA mini-barcode assay, we created a mock community from fecal DNA of three bat species and tested iterations of rarity via next-generation amplicon sequencing; 3) When applied across a landscape, how effective are genetic surveys compared to visual surveys?; and, 4) How does the assay perform in the humid tropics? To test these last two questions about assay performance as a survey tool, we compared genetic and visual surveys in over 40 subterranean roosts across the U.S. Southwest, and in Central America. This study clarifies the limitations of a highly-successful DNA metabarcoding assay for a widely-distributed taxonomic order, and is an example of the types of tests that can be performed for other taxa and assays.

## Materials and methods

### Ethics statement and permits

This study was approved by the Institutional Animal Care and Use Committee (IACUC) at Northern Arizona University (Protocols 07-006-R2, 14–008, and 15–006), the American Museum of Natural History (approval numbers AMNHIACUC-20170403 and AMNHIACUC-20180123), Arizona Game and Fish Department (SP706855), the Navajo Nation (Special Permit 908), and the Forest Department of Belize (permit numbers WL/2/1/17(16) and WL/2/2/18(16)). No bats suffered injury or mortality as part of this study.

### Controlled field experiments: Impacts of time and humidity

To understand the influence of time and humidity on the ability to successfully recover DNA from bat guano and to apply the Species from Feces assay, we selected a cave with high humidity (near Flagstaff, Arizona), a cave with low humidity (on the Navajo Nation in Arizona), and an office environment (FMW’s office at Northern Arizona University) in which to perform a controlled test over 30 months (September 2015 to April 2017). An office was included as part of the study since many biologists store feces in offices with the intention of future genetic analysis. The two caves are sensitive sites so GPS coordinates are not provided. Fresh fecal pellets of uniform size (n = 210) were collected from an *Eptesicus fuscus* roost under the porch of a house in Flagstaff, Arizona, in August 2015. We had previously identified the species using the porch roost via Species from Feces testing prior to collection of these samples. Each fecal pellet was placed into its own 1.5mL tube, which were left with lids open, except for the wet cave for which pellets were placed into small mesh bags due to high humidity in the cave (i.e., condensation would collect in tubes). Time periods were 0 (30 fecal pellets immediately frozen at -80°C), 6 mo (17 pellets per site), 12 mo (17 pellets per site), 18 mo (17 pellets per site), and 30 mo (17 pellets per site). For the dry cave, we placed open tubes into a plastic tub with sides of window screen, and placed the tub at a depth of 180 m in the cave. For the wet cave, we placed the small mesh bags into a stainless steel mesh Armored Outdoor Gear Ratsack Cache food storage bag (Armored Outdoor Gear, Inc., Flagstaff, AZ) and hung the storage bag on a cave wall 150 m into the cave. The office tubes were placed in a cardboard box in a bookshelf in Northern Arizona University’s School of Forestry. At all sites we deployed a HOBO Pro V2 datalogger (Onset, Bourne, MA) to record temperature and relative humidity every 15 minutes.

Upon retrieval, feces were stored at -80°C until DNA extraction for Sanger sequencing. We extracted DNA from each fecal pellet with a QiaAmp Fast Stool Mini Kit (Qiagen, Valencia, CA, USA) following the human DNA protocol. PCRs were performed in 10 μL reactions containing 2 μL undiluted DNA template, 1 μL 10X Mg-free PCR buffer (Invitrogen, Thermo Fisher Scientific, Waltham, MA, USA), 2.5 mM MgCl2, 0.2 mM of each dNTP, 0.4 μM unlabeled primers (SFF_145f, SFF_351r) [3], and 0.3 U/ μL PlatinumTaq DNA polymerase (Invitrogen, Thermo Fisher Scientific, Waltham, MA, USA). We performed PCRs on MJ Research PTC-200 thermocyclers with a first step of 95°C for 10 min, followed by 38 cycles of 60 s at 95°C, 30 s at 60°C, and 30 s at 72°C, and with a final extension step of 72°C for 10 min. Non-template and positive controls were included in all PCRs, and PCR products were assessed on 2% agarose gels. We purified PCR products using the ExoSAP-IT (Affymetrix, Santa Clara, CA, USA) product cleanup protocol, and added the undiluted purified product to a sequencing reaction via BigDye Terminator v3.1 kit (Applied Biosystems, Foster City, CA, USA) following the recommended protocol. An ABI3130 Genetic Analyzer (Applied Biosystems, Foster City, CA, USA) was used to sequence products in both directions, and Sequencher 5.3 software (http://www.genecodes.com) was used for editing sequences. We considered sequencing to be successful when species identity from NCBI’s Basic Local Alignment Search Tool (BLAST) [26] was over 90% across the 202 bp amplicon and when there was no ambiguity among the top 10 hits.

### Tests involving next-generation sequencing

#### Illumina library preparation and sequencing

We tested the metabarcoding capability of the Species from Feces assay with respect to specificity and sensitivity, as well as applicability for species surveys in the U.S. Southwest and the Belizean tropics. The setup for each experiment is described in the sections below. We performed all fecal DNA extractions and next-generation amplicon sequencing as described previously [3]. This preparation results in an indexed library according to [27]. We used a dual indexing strategy, with each sample tagged by two 12 nucleotide-long indices each at 3–4 mismatches away from every other index in the set. This results in a combined index length of 24 nucleotides and 6–8 mismatches from any other dual index. Our benchmarking experiments suggest that this reduces tag jumping to at most 1 in 100,000 reads. While tag jumping could not be fully ruled out, our extremely stringent indexing strategy is designed to minimize its impact. For each experiment, libraries were quantified using KAPA Illumina Library Quantification Kit (KK4933) and indexed samples were pooled in equal concentration. We sequenced pools for each experiment using a 2x300 Illumina MiSeq kit as flow-cell space became available. These runs also included samples that were not part of the current project, which is why we used a kit that sequenced beyond our target amplicon length.

#### Sequence processing and taxonomic classification

We trimmed 5’ primers and adapters for both R1 and R2 reads in cutadapt v1.6 [28]. We merged paired-end reads and filtered in Mothur v1.34.4 [29]. For merged reads, we trimmed overlap (trimoverlap = T) to remove non-biological sequence (i.e., readthrough). This was necessary because we sequenced a 202 bp amplicon on a 2x300 Illumina MiSeq kit, which appended 73 non-biological base calls. We filtered ambiguous bases and set the maximum homopolymer length to 8 bases, which we estimated from homopolymers in our reference library. The merged sequences were filtered to retain a minimum length of 200 bases and a maximum length of 202 bases. We used QIIME v1.9.1 [5] to cluster operational taxonomic units (OTUs) and classify taxonomy. We used pick_rep_set.py to cluster filtered reads to 99% similarity via the UCLUST, furthest-neighbor clustering algorithm [30]. We derived representative OTUs and screened for chimeric sequences de novo using the VSEARCH v1.11.1 uchime_denovo command [31]. Taxonomy was classified for the screened, representative sequences using the RDP classifier [32] with a minimum confidence threshold of 90% for assignment via a reference library of Chiropteran COI sequences described in [3]. We mapped representative OTUs classified to the species level to each sample’s OTU observations to attain per sample read counts.

### Controlled lab experiments: Assay sensitivity and specificity in mock communities

#### Mock community composition

Fecal pellets from *Tadarida brasiliensis* (Mexican free-tailed bat; TABR), *Eptesicus fuscus* (big brown bat; EPFU), and *Corynorhinus townsendii* (Townsend’s big-eared bat; COTO) were used in the mock community preparation. The 3 species’ fecal pellets were of similar size (approximately 2.5mm x 7.5mm). DNA from each pellet (see below for protocol) was extracted separately. *Tadarida brasiliensis*, *Corynorhinus townsendii*, and *Eptesicus fuscus* each alternated as the rare bat in the mixture. Each mixture was prepared at 1:192 μL ratio of a rare bat’s fecal DNA combined with equal parts of the other two bat species’ fecal DNA. These proportions were guided by our fecal collection protocol, in which about 200 fecal pellets are collected as a single sample, with the aim to investigate the question of whether a rare bat species represented by only one fecal pellet in such a sample will be detected using our assay. Thus, for example, the rare *Corynorhinus townsendii* mixture contained ~0.52% *Corynorhinus townsendii* and ~49.74% each of *Tadarida brasiliensis* and *Eptesicus fuscus*. These 3 mixtures were replicated 10 times, for 30 experiments total. To evaluate a less extreme scenario of species rarity in a mixed sample, we repeated this test at a 1:64 ratio of rare bat DNA (or roughly 3 fecal pellets in a pool of 200) combined with equal parts of the other two bat species.

#### Mock community samples—Collection and preparation

We collected fresh feces from captive colonies at Texas A&M (*Tadarida brasiliensis*) and Brown Universities (*Eptesicus fuscus*), and from below an active roost in Sycamore Canyon, Arizona (*Corynorhinus townsendii*). During collection, fecal pellets were deposited individually into 1.5mL microcentrifuge tubes (1 pellet/tube) containing 500 μL of RNAlater (Ambion, Austin, TX, USA) and stored at -80°C until DNA extraction. DNA was extracted from each individual fecal pellet (*Tadarida brasiliensis* N = 247; *Eptesicus fuscus* N = 246; *Corynorhinus townsendii* N = 96) according to the protocol described in [3] and eluted to a final volume of 100 μL. Following DNA extraction, we transferred 75 μL of each DNA extract into standard 200 μL skirted PCR plates. Array plates were used to create the mock community mixtures, with each species represented as rare at 1:192 with the other two species as common in equal proportion. We repeated the tests with our rarest bat species at 1:64.

### Experimental validation: Large-scale application in the arid U.S. Southwest

To demonstrate the large-scale utility of our approach in screening roosts for presence of bat species, we collected guano from 41 abandoned mines in the U.S. Southwest (Arizona, Colorado, Nevada, New Mexico, and Utah) at elevations ranging from 331 to 2,495 m [24]. Abandoned mines in this region outnumber natural caves and are commonly used by bats as roosting habitat [33]. Between June and December 2015, we collected guano samples from the mines, each sample consisting of up to 200 pellets deposited into a 15mL conical containing 7.5mL of RNAlater. We targeted guano pellets that were whole and not disintegrating. Additional collection methods and site details are presented in [24]. We stored samples at 80°C until DNA extraction. At the time of guano collection, mines were visually surveyed for bat species presence, and bats were identified by morphology and body size. We tested the null hypothesis that the number of bat species detected does not differ between survey methods (Mann-Whitney U test [34]). We hypothesized that more species would be detected by the genetic approach because it involves a longer time frame (fecal pellets deposited for months or years) than do visual surveys (a single point in time).

### Experimental validation: Assay performance in the tropics

To determine how the assay performed in the humid tropics, we targeted an area of Orange Walk Province in north-central Belize that is rich in Maya history and where bats have been studied since 1998 [35]. Herrera et al. [36] provided descriptions of the area and of archaeological sites where the bat fauna has been extensively surveyed. This area receives 1500 mm annual precipitation [37], and averages 83.5% relative humidity with highs of 31.7 °C in the January-May dry season [38]. In late April and early May 2017, we collected a single pooled guano sample as described above from each of nine locations in and around the archeological sites. In addition to pellets, a small amount of substrate (soil, sand, crumbled rock) was collected at each site since feces of *Desmodus rotundus* (common vampire bat) are known to be largely liquid [39]. The locations sampled included looter’s tunnels at Ka’kabish Archaeological Site (17.8147 N,-88.73052 W), a tunnel-like archway through ruins of an abandoned brick colonial building at Lamanai Archaeological Reserve (17.75117 N, -88.65446 W), and a sinkhole 5 km southwest of Lamanai Archaeological Reserve. We verified the humidity and temperature in these subterranean tropical systems by placing HOBO dataloggers in two of the tunnels at Ka’Kabish in April and May 2018. These tunnels, constructed by looters searching for artifacts in the unexcavated Maya ruins of the site, are known roost sites that have been occupied by bats for over a decade [36, 40]. As part of ongoing research at the site, we netted bats both at the tunnel entrances using mist nets, and inside the tunnels using hand nets, in both 2017 and 2018. Many bat species in this region have not been COI barcoded (or barcodes are not yet publically available). Therefore, we sequenced 580 bp of COI-5P (primers BEGLCOI in [3]) from specimens of 21 species housed at the American Museum of Natural History (S1 Table) and added them to our reference library before analyzing the amplicon sequencing data [3].

## Results

### Controlled field experiments: Impacts of time and humidity

**Fig 1:**
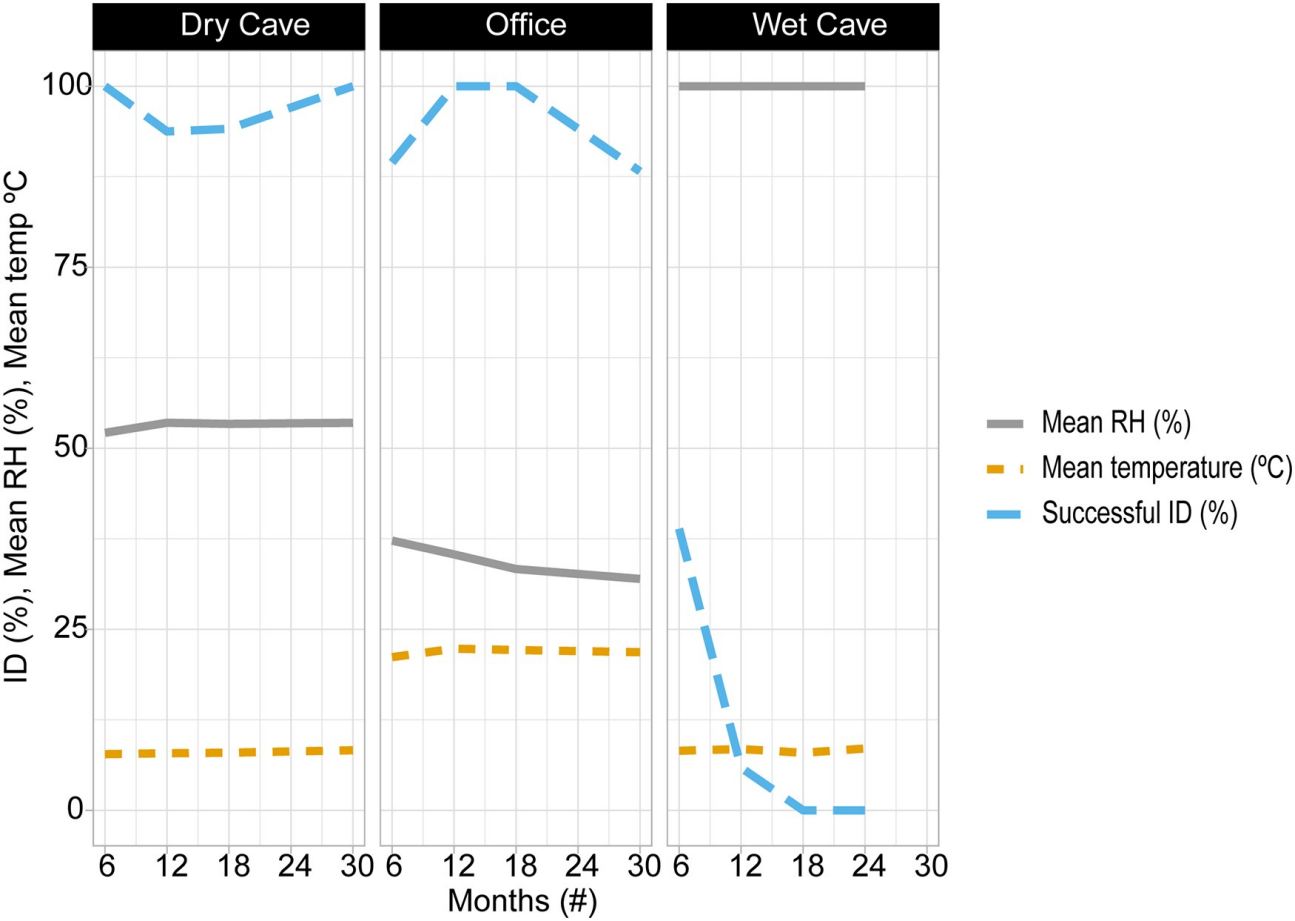
Impact of time and relative humidity on the ability to successfully identify bat species from their guano. Seventeen fecal pellets were tested for each time period in each location.

At time 0, all 30 fecal pellets successfully sequenced. DNA from feces in the dry cave and office experiments were successfully sequenced after 30 months, whereas feces from the wet cave performed poorly at 6 (6 of 17 successful) and 12 (1 of 17 successful) months and all failed at 18 months (Fig 1). Relative humidity at the wet cave was high (100%) and temperature cool (mean 8 °C ± 0.20). At the dry cave humidity averaged 51% (SD = ± 0.55) and temperature was also a consistent 8 °C (± 0.19). The office environment was more variable, with humidity 34.5% (± 2.04) and temperature 22°C ± 0.45 (Fig 1).

### Controlled lab experiments: Assay sensitivity and specificity

**Fig 2:**
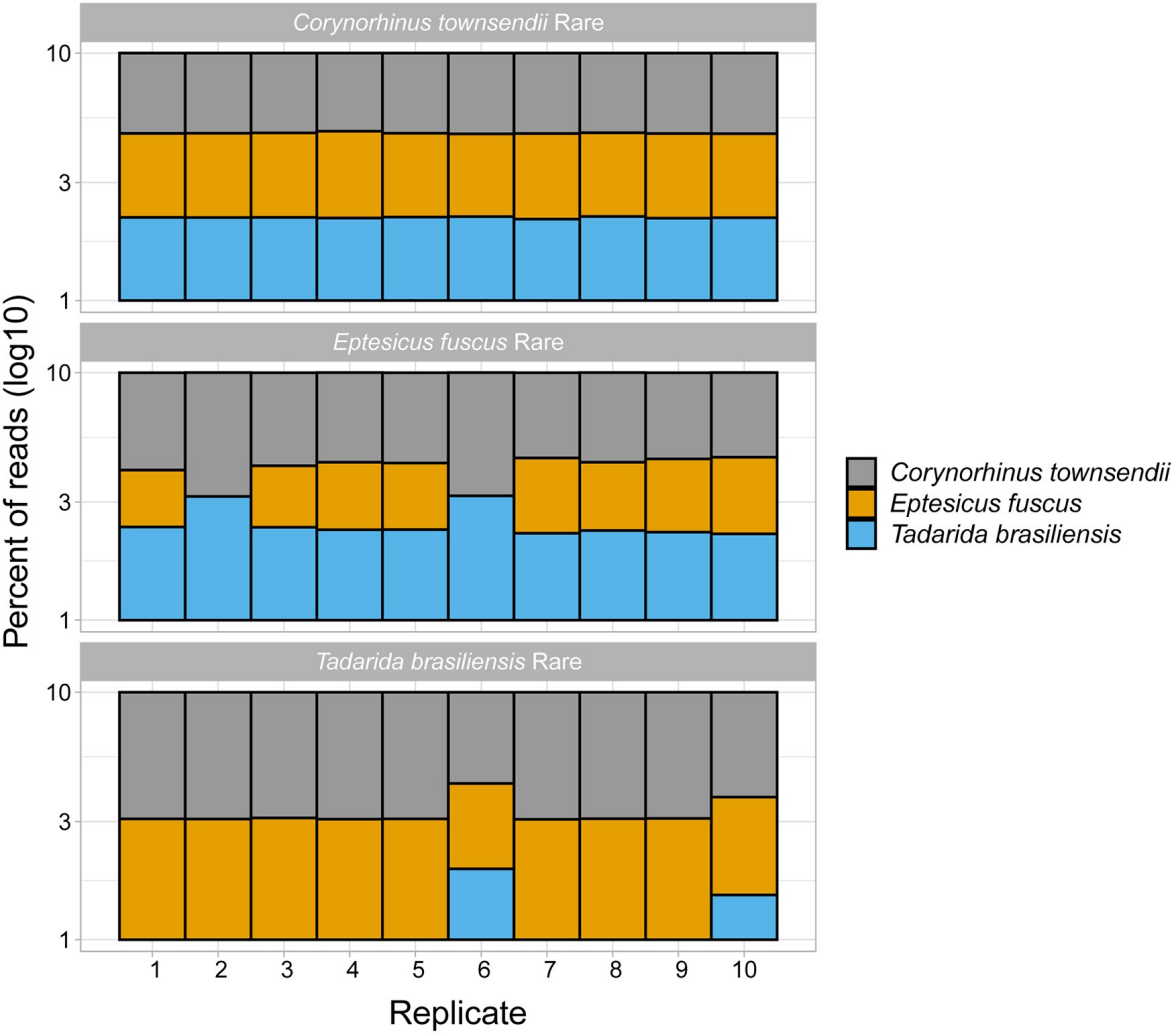
Detection sensitivity of a rare bat species in a mock community of guano from three species. When *Eptesicus fuscus* (EPFU) and *Corynorhinus townsendii* (COTO) were designated rare (at 1:192 ratio of DNA template), they were always or almost always detected (10 of 10 tests and 8 of 10 tests, respectively). *Tadarida brasiliensis* (TABR) was detected in 2 of 10 tests, which suggests a primer bias against this molossid species.

In the mock communities, the designated rare bat species was identified in 20 of 30 mixtures for the 1:192 and 22 of 30 mixtures for the 1:64 ratio tests (Fig 2; SRA accession number SRP223583). Detections at the 1:192 ratio for each bat species as rare are as follows: *C*. *townsendii* 10 of 10 tests, *E*. *fuscus* 8 of 10 tests, and *Tadarida brasiliensis* 2 of 10 tests. At 1:64, *E*. *fuscus* improved to 10 of 10 tests, but *T*. *brasiliensis* remained at 2 of 10. There were no false positives in 29 of the 30 mixtures at both ratios. The mixtures with false positives identified the presence of *Antrozous pallidus*, *Artibeus jamaicensis*, *Artibeus toltecus*, *Myotis evotis*, *Myotis lucifugus*, and *Myotis volans* simultaneously (for 1:192; 2.7% of assigned reads; S2 Table), and *Eumops perotis* (for 1:64; 0.6% of assigned reads; S2 Table), which were species that shared the MiSeq run for other projects.

### Experimental validation: Large-scale application in the arid U.S. Southwest

**Fig 3:**
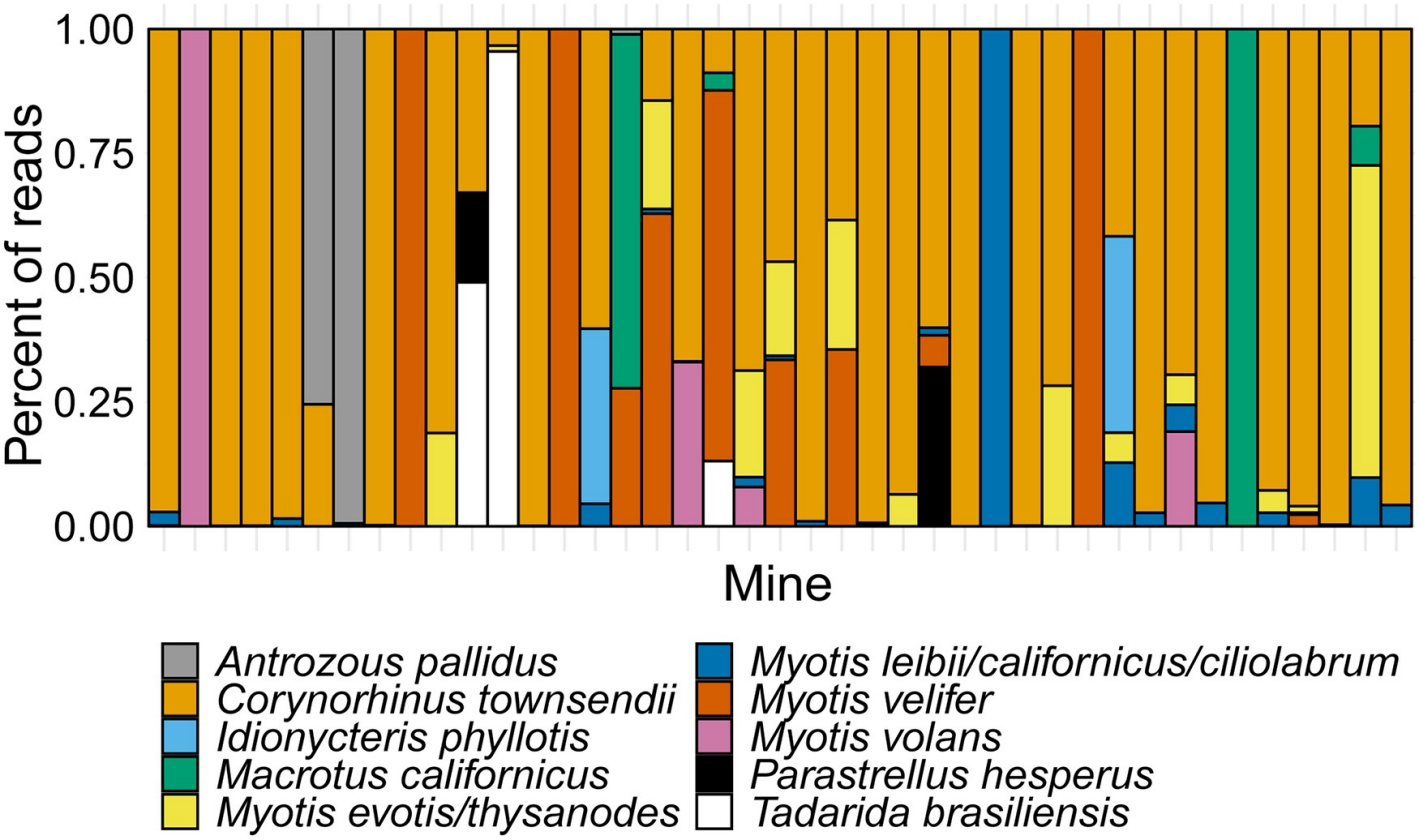
Genetic detection of bat species in 41 mines across the U.S. Southwest, from samples that each contained up to 200 fecal pellets. Species separated by slashes share a DNA mini-barcode.

**Fig 4:**
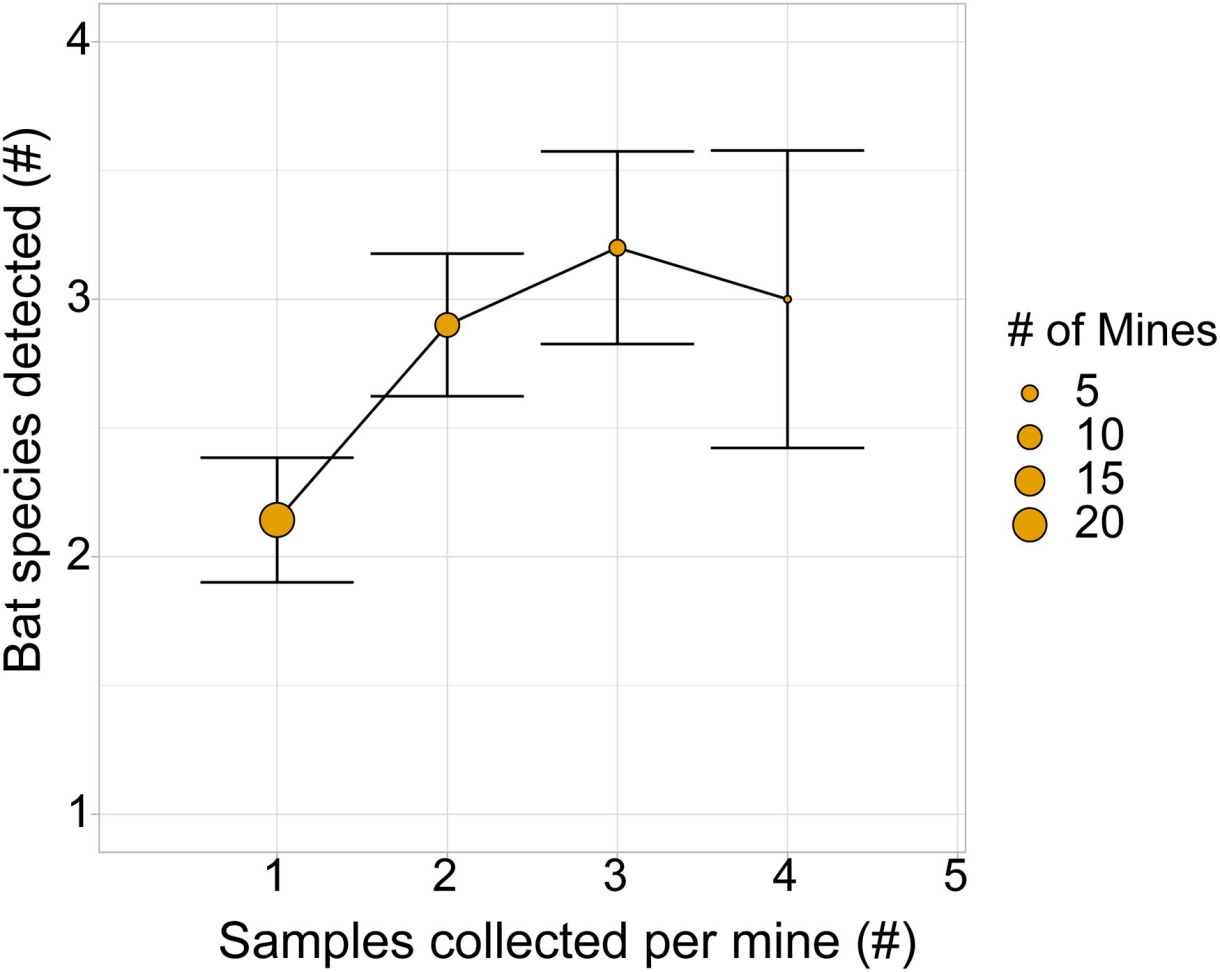
Impact of the number of sample replicates collected in each mine on the number of bat species detected. N is the number of mines in which the indicated number of samples were collected. Error bars are the standard error of the mean for the number of bat species detected in each sampling group. The non-overlapping error bars between one and two pooled samples suggests that collection of two samples will maximize species detection while minimizing sequencing costs.

**Fig 5:**
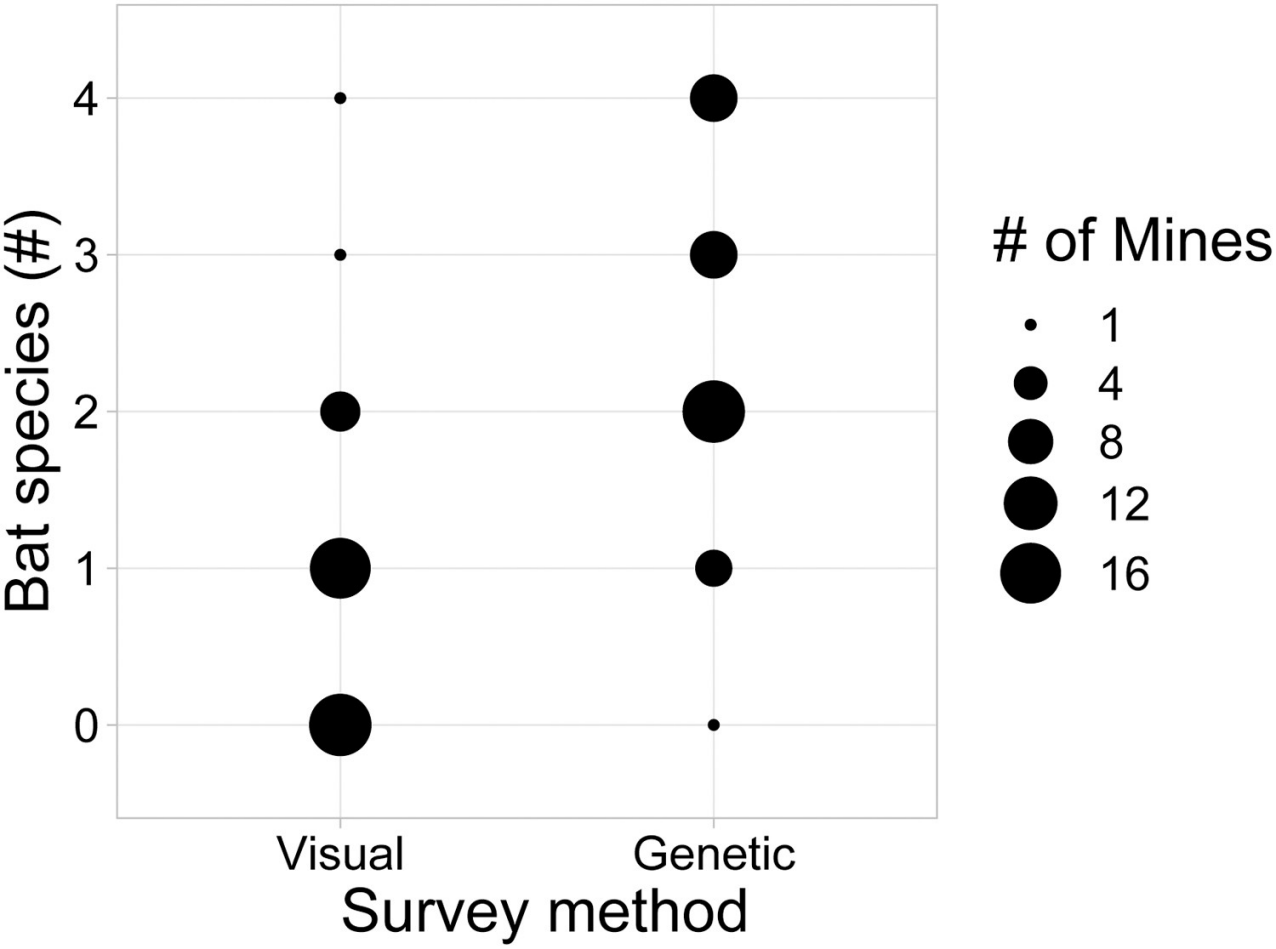
The number of bat species identified in mines from visual surveys vs. genetic screening of guano. Significantly more bat species were detected using genetic methods (z = 5.79, *P* < 0.00001, Mann-Whitney U test).

We detected between 0 and 4 bat species in each mine (Fig 3; SRA accession number SRP187281; S3 Table). Although one sample was sufficient for identifying species in mines, collecting and analyzing two samples provided a more robust index of species richness (Fig 4) while minimizing sequencing costs. Bat species were genetically identified in 98% of mines, and visually identified in 58% of mines (Fig 5), and significantly more bat species were detected using genetic methods (z = 5.79, *P* < 0.00001, Mann-Whitney U test).

### Experimental validation: Assay performance in the tropics

**Fig 6:**
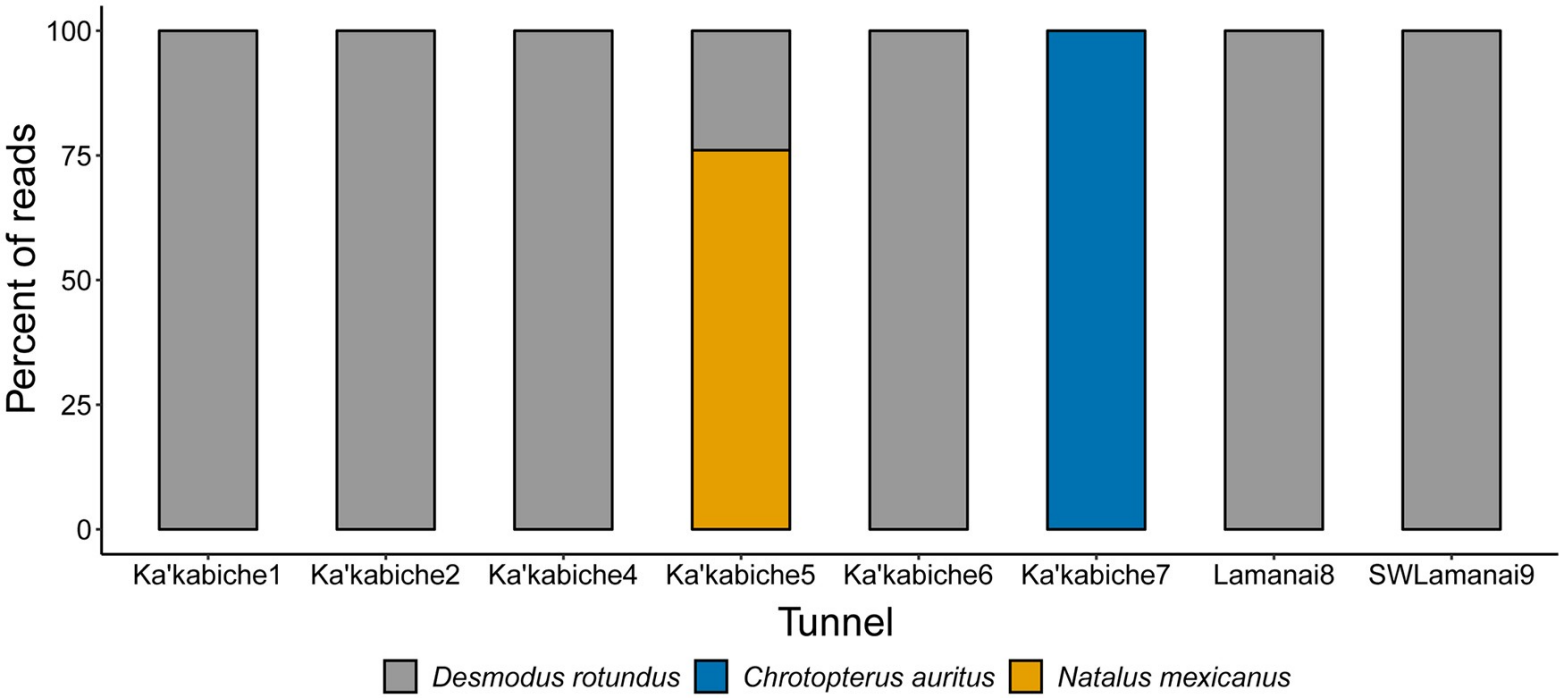
Bat species genetically detected from guano samples collected in tunnels of archeological sites (Ka’kabiche and Lamanai) in Belize.

For 8 of 9 guano samples from tunnels of archeological sites in Belize, we successfully identified bat species on our first amplification attempt (Fig 6; SRA accession number SRP187457; S4 Table). We detected *Natalus mexicanus* (Mexican greater funnel-eared bat), *Desmodus rotundus*, and *Chrotopterus auritis* (big-eared wooly bat), and only 1 species was detected in each tunnel except for Ka’kabiche5 in which both *Natalus mexicanus* and *Desmodus rotundus* were identified. Genetic identification matched the visual species ID (from netting) in 4 tunnels. *Desmodus rotundus* alone was detected in all of the other locations. In two cases, other bat species were known to occasionally use these roosts, but they were not detected in the samples collected. *Desmodus* was the only species detected in samples from the archway through ruins of an old colonial building and in a sinkhole 5 km from the reserve. Although the archway itself was not netted, it is ~25 meters from a large hollow tree that is the roost site for a colony of ~80 *Desmodus rotundus*. *Desmodus rotundus* is a common species in the area and these bats are known to use a variety of different roost sites [36], hence it is likely that they regularly visit both the archway and the sinkhole. Because we designed the Species from Feces primers to detect bats but not arthropod prey, and no other taxonomic groups were intentionally excluded in primer design, we coincidentally detected non-target vertebrates that were either prey for the bats or also inhabited the tunnels. *Bos taurus* (domestic cow) DNA was detected in guano samples from all the *Desmodus rotundus* tunnels, which is not surprising since these bats are thought to feed heavily on livestock from the surrounding area [41]. In guano from the *Chrotopterus auritis* sample, we detected *Ototylomys phyllotis* (the big-eared climbing rat), which may have been a prey item. We also detected *Rhinella marina* (cane toad) in one tunnel, which may have been a resident, and *Gambusia sexradiata* (teardrop mosquito fish) in 8 of 9 tunnels. This fish species is known to inhabit most standing bodies of water in this region, including stock ponds where bats were netted coming to drink in 2018.

## Discussion

Our field and lab tests defined the limitations of the bat Species from Feces assay, revealing factors important to DNA recovery from feces, the power of detection in a mixed species sample, and the utility for roost surveys in arid and tropical areas. In fecal DNA metabarcoding studies, the gold standard is collection of feces while they are fresh in order to maximize DNA recovery [42, 43]. In our study, time was less important than humidity for host DNA recovery from bat feces. At the lower humidity and temperature of the dry cave and office, DNA was reliably PCR-amplified at 30 months, suggesting that it is reasonable to collect samples that may have been deposited several years or more ago if conditions are dry and cool. Even though our office fecal DNA performed well, we do not recommend storage of any type of feces in a desk drawer or other office scenario for future genetic analysis, since relative humidity in many regions is higher or more variable than in the U.S. Southwest. Storage in a freezer after collection is preferable. Finally, the limited ability to PCR-amplify DNA from bat feces in the wet cave at 6 and 12 months and failure at 18 months underlines the degrading effects of humidity on DNA. Moisture, particularly when combined with high temperature, directly damages DNA and fosters microbial growth, thus further degrading DNA [10, 44].

The mock community experiment illustrated that even if a single fecal pellet in a pooled guano sample (typically ~200) is from a different species than the others, it will likely be detected by the Species from Feces assay. However, fewer *Tadarida brasiliensis* sequences when represented by 0.5% and 1.6% of a pooled sample suggests that there may be a primer bias against this mollosid species when its DNA is rare. Primer bias is expected when a single primer set is used across a taxonomic group, and can result in a large variance in read numbers [45]. During Species from Feces assay development we used *in silico* methods along with samples across Chiroptera, finding no inherent non-amplification issues among global barcoded bat species [3]. Hence, although reads for some mollosids may be underrepresented in a mixed sample, false negatives will predominate only when their DNA is exceedingly rare (rarer than 1:64). As for false positives, we found two of 60 mixtures contaminated with reads from species that were on the same MiSeq run for other projects. We attribute this error to imperfect demultiplexing, which results in misassigned sample indices and has been reported in other studies [1, 46]. Our standard Species from Feces protocol merges R1 and R2 reads instead of using only R1 reads in this experiment. Hence, we recommend always using both reads in order to overcome demultiplexing issues and increase accuracy [27, 47]. The assay performed optimally at 1:64, suggesting it may be of value to collect more than one pooled sample from important sites in order to verify genetic identifications. While *T*. *brasiliensis* has been readily detected in lab and field scenarios with this marker and analysis pathway [3, 24], this study’s suggestion of a primer bias against *T*. *brasiliensis* DNA when it is rare suggests that additional samples may be required for studies in which detection of mollosids is critical, such as for endangered species.

Guano sampling for genetic identification of species was an effective means to survey roosts across a landscape, and resulted in more species detected than visual surveys. With ever decreasing analysis costs, the ease of fecal sampling, and the virtual guarantee of detection of at least one bat species per roost, this genetic approach is a real option for understanding which bat species are or were present at roosts. Such information is increasingly important as white-nose syndrome, a bat disease caused by the fungus *Pseudogymnoascus destructans*, continues to spread across the U.S. and Canada [48, 49]. Our finding that more than one pooled sample collected at a roost yielded an additional bat species (from a mean of 2 species with a single sample, to 3 species with an additional sample) again suggests that two samples may be an optimum number for studies that aim to maximize species detections while keeping sequencing costs low.

Despite higher humidity than the arid US southwest, the assay also performed well for surveying roosts in the tropics, with the added benefit of identification of diet of carnivorous and sanguivorous bat species without additional processing or analyses. Notably, species were detected even from non-descript substrate (not fecal pellet form) taken from the tunnel floors, suggesting that visually fresh feces are not necessarily required when a roost has been regularly occupied. Studies of large carnivores have also shown that feces of unknown age can be used for genetic analysis in the tropics [50, 51]. At sites with high humidity this genetic approach may be useful as a survey tool to assess active or recent use, as suggested by impaired sequencing success by 6 months in the wet cave test, whereas in more arid climates the assay can identify roost use over longer time periods.

## Conclusions

The Species from Feces assay was tailored for species-level discrimination across Chiroptera using the characteristically low copy number and degraded DNA in feces. Its efficacy is aided by the taxonomic order including many species with a natural history favorable for DNA recovery in that they roost and commonly defecate in protected features with low/no UV or rain exposure such as caves and mines. This study suggests that 1) DNA in bat guano is recoverable for up to at least 2.5 years if from regions or roosts that are dry and cool, but fresher feces (<6 months) are needed at 100% relative humidity; 2) the assay performs well as a survey tool even in the tropics; and, 3) species that are rare in a mixed species sample will be genetically identified, but two samples from large or sensitive roosts will ensure that all or most species are detected. In an era of rapidly developing DNA metabarcoding approaches, this study is an example of the types of assessments that facilitate understanding of the strengths and limitations of an applied assay, beyond publication of the assay itself (which typically describes primer design, testing, and taxonomic coverage). These assessments include the performance of the study species’ feces across environmental conditions, sensitivity and specificity with the prescribed sampling protocol and rare taxa (when samples are mixtures of species), and the geographic and temporal utility of the assay.

## Supporting information

S1 Table Bat specimens from the American Museum of Natural History that were COI barcoded for this study. (XLSX)

S2 Table Read counts for mock community experiments. (XLSX)

S3 Table Read counts for bat guano samples from mines in the U.S. Southwest. (XLSX)

S4 Table Read counts for bat guano samples from subterranean roosts in Belize. (XLSX)
